# From conception to care: a systematic review of the impact of the climate crisis on reproductive justice

**DOI:** 10.1080/26410397.2025.2576365

**Published:** 2025-10-22

**Authors:** Martina Yopo Díaz, Valentina Gómez Aguirre, Loreto Watkins

**Affiliations:** a Assistant Professor, Instituto de Sociología, Pontificia Universidad Católica de Chile, Santiago, Chile.; b PhD Student, Instituto de Sociología, Pontificia Universidad Católica de Chile, Santiago, Chile; c Researcher, Instituto de Sociología, Pontificia Universidad Católica de Chile, Santiago, Chile

**Keywords:** climate crisis, climate change, reproductive intentions, pregnancy, birth, fertility, care, reproductive justice

## Abstract

The climate crisis poses major challenges to reproductive justice. Climate-related events and natural disasters are severely impacting sexual and reproductive rights as well as the ability of individuals to become parents and care for their children. Through a systematic review conducted using the PRISMA method, this article addresses the impact of the climate crisis on the three core principles of reproductive justice: (1) the right not to have a child; (2) the right to have a child; and (3) the right to parent children in safe and healthy environments. While the review found no empirical studies addressing how the climate crisis impacts the right not to have children, the findings suggest that hurricanes, floods, heatwaves, droughts, and coastal erosion are associated with greater intentions of remaining childless and having fewer children, increasing fetal mortality and preterm births, decreasing new-born sex ratios and birth weight, rising birth risks, declining birth rates, and increasing challenges to parenting and childcare. We argue that the climate crisis fundamentally undermines reproductive justice by preventing individuals from fully and equitably exercising their reproductive rights to have children and to parent in safe and sustainable environments. In doing so, we stress that the climate crisis should be considered when addressing the challenges of reproductive justice and that addressing these challenges requires implementing policies that not only seek to mitigate the effects of climate disruptions but also strengthen the capacity of individuals and communities to adapt to changing environmental conditions, ensuring more sustainable reproductive futures.

## Plain language summary

This article looks at how the climate crisis impacts reproductive health and rights. While our review found no research on how climate change affects people’s right not to have children, it shows that climate-related events can cause important reproductive problems such as higher rates of pregnancy loss, preterm births, and lower birth weights. It also shows that natural disasters such as hurricanes, floods, heatwaves, and droughts are creating uncertain futures and pushing people to remain childless and have fewer children. We argue that the climate crisis threatens reproductive justice by making it more difficult for people to have children and raise them in safe and sustainable environments. This study emphasises that we need policies that not only reduce climate damage but also help people and communities adapt to these changing conditions, ensuring safer futures for families.

## Résumé

La crise climatique pose des défis majeurs à la justice reproductive. Les catastrophes naturelles et les événements liés au climat ont de graves conséquences sur les droits sexuels et reproductifs ainsi que sur la capacité des individus à devenir parents et à s’occuper de leurs enfants. Avec un examen systématique mené selon la méthode PRISMA, cet article étudie l’impact de la crise climatique sur les trois principes fondamentaux de la justice reproductive: (1) le droit de ne pas avoir d’enfant; (2) le droit d’avoir des enfants; et (3) le droit d’élever ses enfants dans un environnement sûr et sain. Bien que l’examen n’ait trouvé aucune étude empirique portant sur l’influence de la crise climatique sur le droit de ne pas avoir d’enfant, les résultats suggèrent que les ouragans, les inondations, les vagues de chaleur, les sécheresses et l’érosion côtière sont associés à une intention plus marquée de ne pas avoir d’enfant et d’en avoir moins, à une augmentation de la mortalité fœtale et des naissances prématurées, à une diminution du rapport de masculinité et du poids des nouveau-nés à la naissance, à une augmentation des risques à l’accouchement, à une baisse des taux de natalité et à une multiplication des difficultés en matière de parentalité et d’éducation des enfants. Nous estimons que la crise climatique sape fondamentalement la justice reproductive en empêchant les individus d’exercer pleinement et équitablement leurs droits reproductifs d’avoir des enfants et de les élever dans un environnement sûr et durable. Ce faisant, nous soulignons que la crise climatique doit être prise en compte dans la résolution des problèmes liés à la justice reproductive, et que s’attaquer à ces défis suppose la mise en œuvre de politiques cherchant non seulement à atténuer les effets des dérèglements climatiques, mais aussi à renforcer la capacité des individus et des communautés à s’adapter aux changements environnementaux, garantissant ainsi un avenir reproductif plus durable.

## Resumen

La crisis climática presenta grandes retos a la justicia reproductiva. Los fenómenos meteorológicos y los desastres naturales tienen un impacto grave en los derechos sexuales y reproductivos, así como en la capacidad de las personas para ser padres y cuidar a sus hijos. Por medio de una revisión sistemática que realizamos utilizando el método PRISMA, este artículo aborda el impacto de la crisis climática en los tres principios fundamentales de la justicia reproductiva: (1) el derecho a no tener hijos; (2) el derecho a tener hijos; y (3) el derecho a criar los hijos en entornos seguros y sanos. Aunque la revisión no encontró ningún estudio empírico que aborde cómo la crisis climática afecta el derecho de no tener hijos, los hallazgos indican que los huracanes, las inundaciones, las olas de calor, las sequías y la erosión costera están asociados con mayores intenciones de no tener hijos o de tener menos hijos, mayores tasas de mortalidad fetal y nacimientos prematuros, menores tasas de masculinidad de recién nacidos, bajo peso al nacer, crecientes riesgos en el parto, tasas de natalidad en descenso y crecientes retos para criar y cuidar a los hijos. Argumentamos que la crisis climática socava fundamentalmente la justicia reproductiva al impedir que las personas ejerzan plena y equitativamente sus derechos reproductivos a tener hijos y criarlos en entornos seguros y sostenibles. Hacemos hincapié en que se debe tomar en consideración la crisis climática al afrontar los retos de la justicia reproductiva, y que para abordar estos retos es necesario aplicar políticas que no solo busquen mitigar los efectos de las perturbaciones climáticas, sino que también refuercen la capacidad de las personas y las comunidades para adaptarse a las condiciones ambientales cambiantes y así garantizar futuros reproductivos más sostenibles.

## Introduction

Climate crisis often refers to the serious problems that are being caused by changes in the climate of the planet, including weather extremes and natural disasters, ocean acidification and sea-level rise, loss of biodiversity, food and water insecurity, health risks, economic disruption, and human displacement.^1^ This crisis is driven by activities like burning fossil fuels for electricity, industry, and transport; deforestation; agriculture; powering buildings; and high levels of consumption, all of which release large amounts of greenhouse gases into the atmosphere, causing the Earth to warm up faster than ever before.^2^ These changes are severely impacting the sustainability of human life by threatening ecosystems, traditional livelihoods, increasing poverty, and exacerbating health vulnerabilities through inadequate access to safe water and sanitation, food insecurity, access to health care and education.^3^

The climate crisis is also posing major challenges for reproductive justice, understood as “(1) the right not to have a child; (2) the right to have a child; and (3) the right to parent children in safe and healthy environments”.^4^ Drawing on the work of feminists of colour, reproductive justice aims to overcome narrow perspectives on reproductive rights that focus mainly on the right to choose to use contraception, practise abortion, and prevent motherhood, by stressing that all persons who reproduce and become parents require a safe and sustainable context for these most fundamental human experiences.^4^ Women of colour activists have further stressed the links between climate justice and reproductive justice by highlighting how structural drivers of climate and environmental injustice lead to harmful reproductive outcomes.^5^ Climate justice, in this context, refers to the fact that those least responsible for causing climate change are often the most severely affected by its consequences, interrogating the social, political, and ethical questions of how climate impacts are differentially distributed.^5^ Because reproductive rights are intertwined to broader environmental issues, climate-related events and natural disasters pose major threats to sexual and reproductive rights as well as to the ability of individuals to become parents and care for their children.

Recent evidence confirms that climate disasters are having profound impacts on sexual and reproductive health.^6,7^ Studies show that environmental changes are linked to declining fertility rates^8^ and sperm counts,^9^ and increasing rates of infertility^10^ and miscarriage.^11,12^ Environment-related risks during pregnancy have been linked to adverse birth outcomes such as low birth weight and preterm birth, adding complexity to parental care in the early stages of child development.^13–15^ Heatwaves, in particular, have been shown to increase the odds of any obstetric complication and pose a major threat to maternal and neonatal health.^16^ Additionally, resource scarcity, such as food and water insecurity, also complicates access to essential reproductive services, including contraception and maternal care.^17^

Concerns about uncertain environmental futures are leading some to reconsider their reproductive intentions,^18–20^ leading to short-term reductions in childbearing.^21,22^ This shift is closely tied to the emotions-driven aspects of reproductive decision making, with environmental considerations influencing whether, when, and why people today decide to become parents – or not.^23^ The climate crisis not only affects family planning decisions but also poses important challenges for those who are already parents. Direct health risks from climate-related events, such as increased heat waves, pose new challenges for parents as they struggle to protect young children who are particularly vulnerable to heat-related illnesses such as exhaustion and dehydration.^24^ This additional stress, combined with growing anxiety about climate change, has intensified concerns about parents’ ability to provide a safe and healthy environment, especially among those who discuss environmental issues with their children.^25^

From an intersectional perspective,^4^ it becomes evident that environmental degradation disproportionately disadvantages the reproductive lives of marginalised social groups, particularly women and pregnant women, children and youth, and Indigenous and Black communities.^5,26^ Pregnant women face higher risks during natural disasters due to barriers in accessing essential health services, compromising their well-being and that of their unborn children.^27^ Amid extreme weather events, pregnant women from Black communities are more exposed to harmful reproductive outcomes such as maternal mortality as well as preterm and low weight births.^5^ Children bear 88% of the health burden attributed to climate change.^28,29^ Children under the age of five are particularly vulnerable to injury, illness, and death during extreme weather events, due to their physiological immaturity and dependence on caregivers who, in crisis situations, are often stressed or incapacitated.^13,30,31^ Moreover, the over-representation of youth in impoverished sectors increases their vulnerability to the effects of climate change.^32,33^

Although there has been growing interest in understanding the impact of the climate crisis on reproductive justice,^34^ the few systematic studies conducted so far have focused only on how long-term changes in temperature and weather patterns affect women’s reproductive health,^35^ maternal, fetal, and neonatal health,^16^ and reproductive decision-making.^36^ Together with these important contributions, a more comprehensive understanding of the impact of the climate crisis on reproductive justice is needed not only to further current knowledge on how climate change impacts affect sexual and reproductive rights from an intersectional perspective but also to enhance social justice amid environmental degradation and contribute to policies ensuring more sustainable reproductive futures.

In this article, we seek to demonstrate how the climate crisis impacts reproductive justice. To address this question, we provide a comprehensive overview of the impact of the climate crisis on sexual and reproductive rights by outlining how climate-related events and natural disasters disrupt reproductive processes ranging from conception to care. Drawing on the findings of a systematic review conducted through the PRISMA method, we argue that the climate crisis fundamentally undermines reproductive justice by preventing individuals from fully and equitably exercising their reproductive rights to have children and to parent in safe and sustainable environments.

## Data and methods

This systematic review aims to understand how the climate crisis impacts reproductive justice. The review followed the guidelines of the PRISMA (Preferred Reporting Items for Systematic Review and Meta-Analyses) 2020 method.^37^ This method has proven to be a useful tool for organising and systematising studies in a variety of areas, including sexual and reproductive rights.^36,38–40^

**Figure 1.**
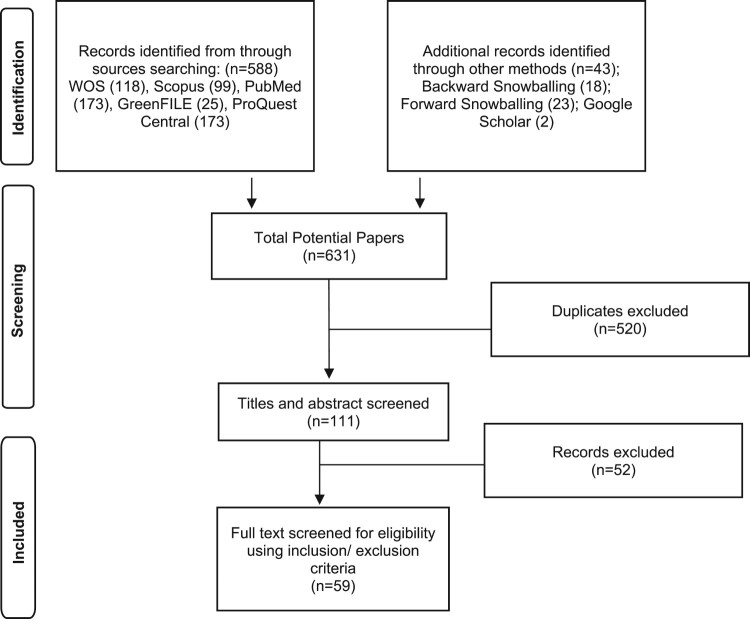
PRISMA diagram of study selection From Page et al.^41^

The data selection process is outlined in Figure 1. First, we conducted literature searches in relevant databases (Web of Science, Scopus, GreenFILE, ProQuest, PubMed). During this search, we focused on subjects related to climate crisis and reproductive justice by using the following query: “climate crisis” OR “climate change” AND “contraception” OR “contraceptives” OR “contraceptive methods” OR “birth control” OR “family planning” OR “pregnancy” OR “miscarriage” OR “stillbirth” OR “pregnancy loss” OR “voluntary interruption of pregnancy” OR “pregnancy termination” OR “abortion” OR “prenatal care” OR “birth” OR “perinatal” OR “neonatal” OR “maternal health” OR “care” OR “sexual rights” OR “reproductive rights” OR “sexual health” OR “reproductive health” OR “sexual and reproductive rights” OR “sexual and reproductive health” OR “reproductive health services” OR “fertility” OR “childbearing” OR “infertility” OR “reproduction” OR “reproductive justice”. Then, we searched for relevant articles by manually searching the reference list of selected articles (backward snowballing) and reviewing the publications that cited them (forward snowballing).^42^ Finally, Google Scholar was used to ensure a comprehensive search of all relevant literature addressing the impact of the climate crisis on reproductive justice. The full electronic search strategy for sources consulted, including all filters and search terms, is provided in Supplementary File 1. The search was conducted during 16 May and 6 June 2024.

Only empirical studies using primary or secondary data published between 2014 and 2024 that explicitly referred to the climate crisis or climate change in the title were included. The aim was to review the most up-to-date literature on the subject. We excluded theoretical articles, book chapters, theses, systematic reviews, theoretical reviews, meta-analyses, narratives, monographs, commentaries, newspaper articles, editorials, calls for papers, letters to the editor, viewpoints, and reports. We also excluded articles referring to plant or animal reproduction and all articles that were not published in English. The search was not restricted by geographic location to allow for critical reflection on scientific production of the impact of the climate crisis on reproductive justice across countries and geographical areas.

After applying the eligibility criteria, 118 articles were selected from Web of Science, 99 from Scopus, 173 from the PubMed database, 173 from ProQuest Central, and 25 from the GreenFILE database. Additionally, another 43 articles were added through backward snowballing, forward snowballing and a search in Google Scholar. The search allowed a first selection of 631 articles, which after checking for duplication (*n* = 520) resulted in 111 articles. The research team then conducted an independent qualitative review to determine the relevance of the articles. Each researcher reviewed the list of articles and independently assessed the titles and abstracts to decide whether each article should be included in the review. A joint triangulation of criteria was then carried out and consensus was reached on the final list of articles to be included in the review. From the total number of articles assessed (*n* = 111), nearly half (*n* = 52) were excluded for not complying with the eligibility criteria, resulting in a total of 59 empirical articles addressing the impact of the climate crisis on reproductive justice.

Although the search terms included a wide range of concepts related to sexual and reproductive health, the inclusion of studies was based on an in-depth qualitative assessment of whether the impacts described could be interpreted through the lens of reproductive justice. Specifically, we examined whether the effects of extreme climate events could be linked to one or more of the three core principles of reproductive justice: (1) the right not to have a child, (2) the right to have a child, and (3) the right to parent in safe and healthy environments. This categorisation was made after reviewing not only titles and abstracts but also the results and discussions of each study. Therefore, only studies whose findings could be explicitly connected to the reproductive justice framework were included.

**Figure 2.**
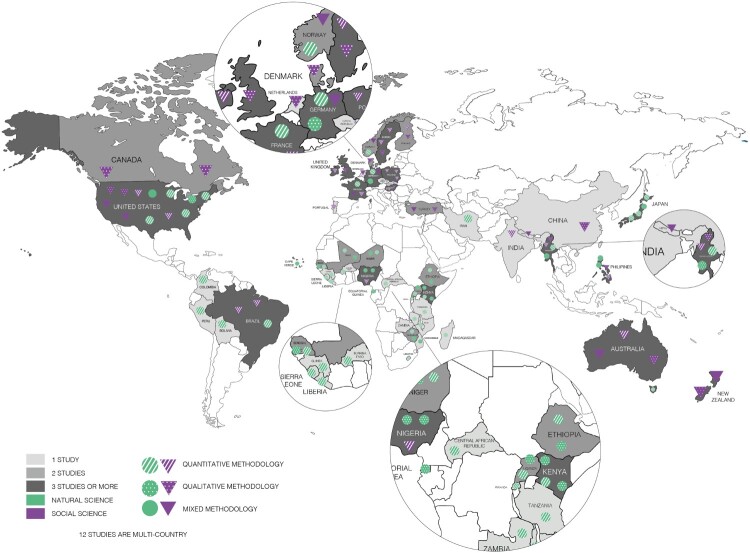
Characterisation of selected studies

A map plotting relevant information of the selected articles is presented in Figure 2. The articles selected for this review were mainly published between 2020 and 2024 (*n* = 39), showing a growing interest in studying these issues in recent years. Of the total number of articles selected, only a few refer to climate crisis (*n* = 2), with the term climate change being more frequently used (*n* = 57). The selected articles cover different disciplines, ranging from the social sciences (*n* = 27) to medicine and health science (*n* = 21) and to environmental sciences (*n* = 11).

The selected studies include high-income countries as well as low- and middle-income countries, with the majority originating from high-income countries such as the United States (*n* = 9), Japan (*n* = 3), Sweden (*n* = 3), Canada (*n* = 2), New Zealand (*n* = 1), Denmark (*n* = 1), and the United Kingdom (*n* = 1), among others. Low- and middle-income countries in the review are represented by Bangladesh (*n* = 3), Brazil (*n* = 2), Philippines (*n* = 3), Kenya (*n* = 2), Iran (*n* = 1), Nepal (*n* = 1), and African countries such as Rwanda, Uganda, Zimbabwe. In addition, there are some global (*n* = 3) and multi-country (*n* = 14) studies. The methodological approach of the reviewed studies was mainly either quantitative (*n* = 32) or qualitative (*n* = 22), with few using a mixed-methods approach (*n* = 5).

The selected studies were screened for relevant information, which was extracted into a detailed matrix. Data extraction was performed manually in Microsoft Excel by one researcher, who organised the extracted information into a structured matrix. Two additional researchers subsequently reviewed the matrix for accuracy and completeness. The matrix was designed to capture the most relevant elements for synthesising the studies, including details on the article (title, authors, year of publication, abstract, journal, and discipline), aims of the study, place where the study was conducted (continent, country, region/city), methods (quantitative/qualitative/mixed, data collection techniques, sample size and composition), dimensions of the climate crises, dimensions of reproductive justice, and key findings.

Given the variation in exposure and outcome measurement, findings were outlined through a narrative synthesis^43^ aiming to characterise the impact of the climate crisis on specific dimensions of reproductive justice spanning from conception to care. To minimise potential bias, the Critical Appraisal Tools (CATs) developed by Aromataris et al^44^ were used.

## Results

Our systematic review aimed to understand the impact of the climate crisis on reproductive justice by outlining how this phenomenon affects (1) the right not to have a child; (2) the right to have a child; and (3) the right to parent children in safe and healthy environments. However, our review did not find any empirical studies examining how the climate crisis affects the right not to have children. Despite a thorough search of studies addressing topics such as contraception, birth control, family planning, abortion, and sexual and reproductive rights, we found no empirical studies using primary or secondary data to outline how climate-related events affect the right not to have children – a fundamental dimension of reproductive justice. This finding reveals a critical gap in empirical evidence on the intersection of the climate crisis and reproductive justice. It also underscores the urgent need for further research into how extreme weather disrupts sexual and reproductive rights, particularly regarding access to contraception and abortion. We return to this critical issue in the Discussion section.

Our systematic review did identify a growing body of research documenting how the climate crisis disrupts the right to have children and to parent in safe and healthy environments. The findings indicate that climate-related events such as hurricanes, precipitations, floods, heat waves, droughts, sea-level rise, and coastal erosion are associated with greater intentions of remaining childless and having fewer children, increasing fetal mortality and preterm births, decreasing new-born sex ratios and birth weight, rising birth risks, declining birth rates, and increasing challenges to parenting and childcare. These findings suggest that the climate crisis fundamentally undermines reproductive justice by hindering the rights of individuals to have children and parent in safe and sustainable environments.

### The right to have children

In this section, we discuss the findings of studies addressing the impact of the climate crisis on the right to have children. Particularly, we discuss how climate-related events affect reproductive intentions, prenatal outcomes, and fertility rates. Of the reviewed studies (*n* = 59), 33 articles address the impact of the climate crisis on the right to have children. Geographically, these studies focus mainly on Europe (*n* = 10), Asia (*n* = 7), North America (*n* = 7), and Latin America (*n* = 4), with only one study representing Africa (*n* = 1). Quantitative approaches dominate methodologically (*n* = 23), with fewer studies employing qualitative (*n* = 6) or mixed-method (*n* = 4) designs. Most papers focus on individuals of reproductive age (*n* = 20) or on pregnant women and new-borns (*n* = 11), with an important lack of diversity in terms of race, ethnicity, and gender identity.

#### Reproductive intentions

The climate crisis affects the reproductive intentions of men and women of reproductive age with and without children, hindering their right to have children and become parents. Concerns about uncertain environmental futures are associated with a reduced desire to have children and a significant increase in intentions to remain childless.^19,45–47^ This trend is supported by multiple studies, especially those conducted in Europe, where high concern about climate change correlates with lower fertility intentions, particularly among those without children.^19,20,45^

For example, a study conducted in Finland, Estonia, and Sweden showed that concern about climate change is negatively associated with fertility intentions.^19^ People who are very concerned about climate change are more likely to have intentions to remain childless and are less likely to plan having children compared to those less concerned about climate change. This effect is particularly noticeable among younger age groups, specifically those aged 18–24 and 25–34. Those who do not have children and do not want to have children in the future report climate change as an important reason twice as often as those who do want to have children in the future or already have children.^48^

Fears over uncertain futures caused by climate change lead women with and without children to reconsider their reproductive decisions, including having fewer children or turning to alternatives such as adoption.^45,47,49,50^ Recent experimental research supports these findings, showing that exposure to pessimistic climate change narratives significantly reduces fertility desires among young adults. A study conducted with university students in Belgium and Italy found that participants who read a pessimistic scenario about the climate crisis were more likely to report low fertility desire.^51^ Research has shown that men and women’s childbearing decisions often focus on the impact of future children on the planet and concerns about contributing to overpopulation.^20^

While some parents and parents-to-be are critical of the idea that climate change is an individual responsibility,^46^ others see the decision not to have children as the greatest individual contribution they can make to mitigating environmental impacts.^20^ For example, Schneider-Mayerson & Leong^46^ found that 59.8% of their US respondents were “very” or “extremely” concerned about the carbon footprint of procreation. This group sees overpopulation and overconsumption as the main causes of climate deterioration and sees forgoing children as an effective way to reduce their carbon footprint. Similarly, in a study conducted in Hungary,^50^ several participants chose to have fewer children, citing environmental concerns; however, they also noted that they expected future generations to adopt more environmentally conscious lifestyles, potentially contributing to climate solutions.

#### Fertility and prenatal health

The climate crisis also extends its impact to fertility by causing important variations in birth rates. Studies reveal that temperature increases and days with high mean temperatures have a negative impact on fertility, leading to declines in birth rates.^52–54^ The impact of increased temperatures on fertility is often mediated by other factors such as reproductive health, crop production, food security, and infant mortality.^53^ Heat waves appear to be associated with reduced fertility, possibly due to biological factors such as reduced sperm quality and changes in women’s menstrual cycles caused by heat stress. In addition, extreme heat has been associated with shorter gestation periods and increased infant mortality, which affects overall fertility in regions with hot climates such as Brazil.^54^ While heightened heat has a negative impact on birth rates in the short term, it could also have a neutral or positive effect in the long term,^52,53^ suggesting that communities may gradually adapt to extreme climatic conditions or modify their reproductive behaviours according to environmental fluctuations.

In Brazil, findings show that both gradual and abrupt increases in temperature are related to fertility decline, consistent with previous research in the United States, Europe and Asia.^54^ However, another study conducted in Bangladesh shows ambivalent evidence regarding the impact of the climate crisis on fertility; while an increase in maximum temperatures has a negative impact on fertility in the short term, in the long term this impact tends to be positive.^52^ Specifically, temperatures above 26.6°C [80°F] were documented to cause a marked decrease in birth rates 8–10 months later, followed by a partial recovery. Possibly, an increase in maximum temperature benefits crop production, which in turn reduces infant mortality and consequently decreases fertility.

Increases in precipitation also impact fertility, although there is conflicting evidence regarding the effect of rises in rainfall on birth rates. While precipitation has been found to have a direct and positive effect on fertility rates,^53^ it has also been found to be associated to a decline in births.^54^ A study in Brazil, for example, examines how extreme weather conditions, such as heat waves and heavy rainfall, impact the number of births and fertility, particularly during the Zika epidemic.^54^ Heavy rainfall exacerbated health and infrastructure challenges – such as flooding, inadequate drainage and the spread of mosquito-borne diseases such as Zika – which increased risks to maternal and fetal health and discouraged pregnancy. The study suggests that during periods of extreme rainfall, the combination of poor infrastructure and increased risk of disease transmission had a deterrent effect on fertility, as couples delayed pregnancy to avoid adverse health consequences. In addition, in rural areas, increased rainfall was associated with economic stress, potentially reducing fertility due to resource scarcity and reduced agricultural yields.

A few recent studies conducted in Iran and Japan further address the impact of extreme weather events on fetal health and well-being during pregnancy. These studies suggest a direct and significant relationship between the increase in average annual temperatures and the surge in fetal mortality rates, particularly in male fetuses, resulting in decreasing male births and new-born sex ratios.^55,56^ Studies conducted in Japan demonstrate that environmental stress caused by rising temperatures significantly increases fetal mortality, particularly among male fetuses, leading to a decline in the proportion of male births.^55,57^ This evidence suggests that environmental stress caused by climate change could be altering key biological mechanisms, such as Y sperm viability and hormonal conditions during gestation, leading to an imbalance in the sex ratio at birth.^57^

### The right to parent children

In this section, we discuss the findings of studies addressing the impact of the climate crisis on the right to parent children in safe and sustainable environments. Particularly, we discuss how climate-related events and natural disasters negatively impact maternal and child health, postnatal care, and childcare. Of the 59 reviewed studies, 26 examine the impact of the climate crisis on the right to raise children in safe and sustainable environments. Geographically, the studies focus on Africa (*n* = 7), Oceania (*n* = 5), Asia (*n* = 5), North America (*n* = 4), and Europe (*n* = 3), with four spanning multiple continents. Methodologically, qualitative approaches dominate (*n* = 16), followed by quantitative (*n* = 9) and a single mixed-methods study. Regarding study populations, the majority focus on children and young people (*n* = 7), followed by studies on the general population (*n* = 2), older adults (*n* = 2), and vulnerable communities (*n* = 2), with an important lack of diversity in terms of gender, race, and ethnicity.

#### Maternal and child health

The reviewed studies show that the climate crisis has a critical impact on pregnancy outcomes and maternal and child health. A rise in mean annual temperature as well as exposure to additional days with extreme heat during pregnancy are associated to reduced birth weights and increasing risk of low birth weights.^55,58^ Since the 1970s, it has been shown that birth weight has decreased by approximately 200 grams in singleton pregnancies in both boys and girls in Japan, and this reduction is significantly inversely associated with differences in mean annual temperature.^55^ Similarly, a study in Hungary showed that exposure to an additional day with a mean temperature above 25°C (77°F) reduces birth weight by 0.46 grams, while the impact of a day between 20°C and 25°C (68°F and 77°F) is – 0.34 grams. Cooler temperatures below the omitted category (15–20°C; 59–68°F) seem to have positive, but insignificant, effects on birth weight. Consequently, average birth weight is projected to decrease, while the prevalence of low birth weight is likely to increase in the following years.^58^

Studies conducted in middle-income countries also show that increased average annual temperatures and exposure to extreme heat during pregnancy reduce birth weight and increase the risk of low birth weight. A study conducted in Brazil by Pereda et al^59^ found that heat and cold waves, as well as extreme precipitation, increase the incidence of low birth weight, particularly in vulnerable regions such as the northeast of the country. Climate change is projected to exacerbate this trend, with an expected increase of up to 3171 additional cases of low birthweight annually by 2041–2070, reflecting a global trend of deteriorating birth outcomes due to climate change. Another study found that in Brazil, Colombia, and Peru, temperature variability equivalent to one standard deviation of the historical local mean during pregnancy is associated with a reduction in new-born birth weight of about 20 grams.^60^ The same temperature variability increases the probability of low birth weight by 0.7%.

Heat waves exacerbated by climate change also increase the risks of fetal stress, preterm birth, and health complications for mothers and new-borns.^59,61,62^ Babies born during periods of extreme heat not only face a higher proportion of health complications, but there is also a marked increase in fetal stress and meconium aspiration syndrome.^56,61^ In addition, mothers are more likely to suffer from adverse health conditions, such as pregnancy-related hypertension and pre-eclampsia.^56^

Droughts and decreased rainfall are also critical factors impairing maternal and child health, particularly in vulnerable regions. Diminished precipitations during pregnancy have been identified as key modulators of food security, negatively affecting maternal health and birthweights due to decreased availability and access to food.^63,64^ This food insecurity, exacerbated by extreme weather conditions, has been recognised as a key determinant of infant health at birth. Low relative humidity and lack of precipitation also negatively impact the health of new-borns, increasing the risks of neonatal deaths and low birth weights.^59^

#### Birth and postnatal care

Climate-related events and natural disasters driven by the climate crisis have emerged as significant drivers of displacement, forcing millions of people to leave their homes each year. Displacement poses important risks to maternal and new-born health during birth. A study conducted in rural Bangladesh found that mothers from households displaced by floods or riverbank erosion are less likely to give birth in a health facility compared to mothers from non-displaced households, despite sharing socio-cultural similarities and residing in the same region.^65^ At the same time, displaced mothers are also less likely to use postnatal care services, especially those provided by skilled providers. The study also reveals that mothers in displaced households face severe socio-economic disadvantages that influence their access to health care, contributing to lower utilisation of skilled medical services, increasing the risk of maternal and neonatal mortality.

Pregnant women also face increased risks during extreme weather events due to the unavailability of medical services and transportation difficulties. Another study conducted in Bangladesh also showed that pregnant women face serious difficulties in getting regular prenatal check-ups due to unavailability of doctors and closure of health facilities.^27^ The lack of qualified medical personnel for regular prenatal check-ups and the difficulty of transporting women with birth complications to healthcare facilities increase the likelihood of giving birth in unsafe conditions as well as the risk of severe complications and maternal mortality.^27^ In such circumstances, many women give birth in unsafe conditions or in transit, and delays and difficulties during transportation increase the risks of complications and death.

Other studies also confirm that climate change affects access to reproductive health services, particularly in climate-vulnerable regions of low-income countries. For example, a study conducted in Nepal^66^ reveals that 64% of women surveyed in flood-prone river basins reported disruptions to antenatal and postnatal care due to extreme weather events, while 82% feared being unable to protect their children during or after climate disasters – fears that shape future reproductive decisions. In the study, women indicated that their willingness to have children was influenced by the difficulty of ensuring safe pregnancies and the risk of maternal and neonatal mortality due to difficulties accessing healthcare during monsoons and landslides.

#### Parenting and childcare

The climate crisis is significantly impacting various aspects of family life, particularly affecting parenting and caregiving. An increasing amount of research has highlighted the emotional burdens that climate-related events place on both parents and children. These burdens range from trauma associated with floods, droughts, and wildfires to more persistent issues like eco-anxiety, which is a chronic fear of environmental destruction.^67^

Some studies specifically examine how emotional distress related to climate change affects parenting plans and practices, especially among women. For instance, a study conducted in Canada found that young women’s views on parenting were heavily influenced by eco-anxiety and concerns about an uncertain environmental future.^49^ Participants shared significant worries about the quality of life their children might face in a world affected by pollution, climate instability, and increasingly frequent natural disasters. Feelings of helplessness regarding forest fires, poor air quality, and extreme weather events were common, along with general fears about raising children in a threatened and deteriorating environment.

While some individuals express concern about the carbon footprint associated with having children, these environmental considerations are often overshadowed by deeper existential anxieties regarding the world their children will face. A large-scale survey conducted in the United States found that 96.5% of respondents felt either “extremely worried” or “very worried” about the climate impacts on their current, expected, or hypothetical children.^46^ These emotions extend beyond rational assessments of environmental risks; they encompass feelings of grief, fear, and anticipatory loss. In fact, 6.3% of parents reported feeling some regret about having children, largely driven by a sense of hopelessness and despair about the future.^47^

These emotional concerns are not limited to prospective or new mothers. Studies suggest that the climate crisis is also affecting the emotional experience of fatherhood. Fathers who take on significant caregiving responsibilities frequently report heightened concern about their children’s future in light of climate threats.^68,69^ These fathers often express feelings of sadness, anxiety, and despair, particularly as they confront their perceived inability to protect their children from rising temperatures, natural disasters, and a lack of political action to address these challenges.^70,71^ Gaziulusoy^71^ notes that for many parents, this sense of helplessness is accompanied by feelings of compromised moral integrity and guilt, as they struggle to reconcile their parental role with their environmental values.

The emotional burden on parents often extends to their children. Children and teenagers are increasingly aware of climate risks and frequently internalise their parents’ concerns. This can lead to shared eco-anxiety within families, as younger members express feelings of fear, sadness, anger, and confusion about the future of the planet.^72^ These emotions can strain family dynamics and add further stress to already burdened caregivers. As climate-related anxieties increase, research has linked them to broader mental health issues among children and adolescents, including symptoms of depression, anxiety, and emotional dysregulation.^73,74^

In addition to these psychological impacts, the climate crisis directly threatens children’s physical health, complicating parenting and caregiving. Extreme weather events – such as floods, heatwaves, and storms – can disrupt access to healthcare and basic services, leading to worse health outcomes for children. For example, flooding can result in poor sanitation and outbreaks of infectious diseases like diarrhoea, while heatwaves can increase the risk of dehydration and respiratory problems.^27,54^ These environmental disruptions force parents, particularly mothers, to adapt their caregiving practices under stressful and unpredictable conditions. Many are compelled to prioritise their children’s immediate safety and health in situations where clean water, adequate shelter, and reliable healthcare may be compromised.^27^

## Discussion

The climate crisis is having and will continue to have direct impacts on reproductive justice. The findings of our systematic review clearly outline how climate-related events and natural disasters are severely disrupting reproductive processes ranging from conception to care, including reproductive intentions, pregnancy, birth, fertility, and parenting. Based on this evidence, we argue that the climate crisis is fundamentally undermining reproductive justice by preventing individuals from fully and equitably exercising their reproductive rights to have children and to parent in safe and sustainable environments.

The findings of our review reveal that the climate crisis significantly influences people’s reproductive intentions, often leading to reduced desires to have children. Concerns about long-term changes in climate patterns are associated with important constraints in reproductive decision making and increasing intentions of remaining childless and having fewer children due to fears about an uncertain future. For those who desire to have children, climate-related events can be an obstacle, as unsafe conditions and unpredictable futures make it increasingly challenging to fulfil these reproductive aspirations. In doing so, the climate crisis is severely impairing reproductive autonomy by restricting the right of individuals to have children and become parents.

Increased temperatures and decreased rainfall also have an important effect on pregnancy and pregnancy outcomes, characterised by an increase in fetal mortality and preterm births, and a decrease in new-born sex ratios and birth weight. Floodings and the erosion of riverbanks, as well as their direct effect on population displacements, also hinder access to reproductive health services before, during, and after childbirth. These events increase the risks of neonatal mortality during unsupervised home births, infringing on the right to conceive, gestate and give birth in safe and healthy conditions. Extreme climate events associated with changes in temperature and precipitation also affect fertility leading both to increases and decreases in birth rates and deteriorating the health and well-being of populations. Additionally, the climate crisis contributes to growing physical and mental health struggles among children, young people, and parents. These disruptions add to the mental strain on caregivers, who must confront the challenges of maintaining their children’s well-being amidst increasing environmental deterioration.

An intersectional perspective is vital to account for that fact that while the climate crisis negatively affects the reproductive lives of all, its impact varies greatly according to structural inequalities. Communities suffering from poverty, precarity, and displacement^59,63,65^ as well as children and pregnant women,^27,75^ are particularly vulnerable to extreme weather conditions and environmental disasters. Babies are at higher risk of low birth weight and health complications,^55,57,61^ while children disproportionately suffer from the emotional burdens of eco-anxiety.^72^ Pregnant women in rural flood-prone areas often face dangerous barriers to accessing reproductive healthcare, leading to increased maternal and neonatal mortality rates during disasters, as limited infrastructure and delayed emergency responses hinder safe births.^27^ Particularly in low-income communities, pregnant women often lack the resources to adapt and protect themselves from climate-related health impacts,^75^ which make them more vulnerable to the negative effects of environmental shocks.

Interestingly, individuals do not just passively experience the adverse consequences of long-term changes in climate patterns but also develop strategies to mitigate the impact of climate-related events in their reproductive lives and create more sustainable futures. These strategies are diverse and span from employing various forms of waste reduction,^50^ to building collective relationships and community engagement to strengthen the adaption to climate challenges.^76^ Furthermore, changes between the short-term and long-term effects of climate change on human reproduction indicate not only that individuals and communities modify their reproductive behaviours according to environmental fluctuations, but also that they can adapt to extreme climatic conditions.^52,53^ These strategies demonstrate reproductive resilience amidst environmental deterioration and should be further explored by future research in this field.

While providing robust evidence to document the impact of the climate crisis on the right to have children and parent in safe and sustainable environments, our review also identified a significant lack of empirical evidence on how the climate crisis affects the right not to have children – an essential principle of reproductive justice. This absence highlights a critical research gap at the intersection of climate change and sexual and reproductive rights, particularly in relation to access to abortion and contraception.^38^ Interestingly, the few studies addressing the relationship between climate change and the right not to have children have focused on the impact of contraception and abortion on environmental sustainability. For example, research has highlighted how contraception and abortion can help mitigate climate change by reducing unplanned pregnancies and slowing population growth.^77,78^ Other studies have raised concerns about the environmental impact of contraceptive waste, including water contamination from synthetic oestrogens in hormonal methods.^7^

Although not referring to the climate crisis, there is evidence that climate-related events can lead to mass displacement and the collapse of healthcare infrastructure, limiting access to contraception and abortion.^79,80^ In Bangladesh, for instance, extreme weather events such as floods and cyclones have severely limited access to sexual and reproductive health services. This has led to increased rates of unintended pregnancies and maternal health complications, especially among rural and economically disadvantaged women, compromising their bodily autonomy and right to make informed reproductive choices.^81^ Additionally, gender-based violence tends to surge after natural disasters, increasing the demand for contraception and sexually transmitted infections (STI) prevention.^79,82^ This evidence suggests that it is vital to consider how environmental degradation affects reproductive autonomy and human rights, including the right not to have children.

### Implications for policy

Reproductive justice is at the forefront of the challenges posed by the climate crisis. It is then vital to ensure that sexual and reproductive rights are central considerations when tackling issues related to climate-related events, natural disasters, and environmental deterioration.^6^ Overcoming this challenge demands a comprehensive approach that integrates reproductive issues with climate adaptation strategies to strengthen community resilience.^83^ Consequently, policies aiming to tackle the climate crisis should not only aim to mitigate the causes and manifestations of global disruptions in climate patterns but also prioritise equitable access to reproductive healthcare and support community resilience in adapting to changing environmental conditions.

It is essential to develop support programmes that help families during pregnancy and parenting in areas directly affected by climate change, including financial assistance, access to adequate nutrition, and mental health services to manage the stress and anxiety caused by climate uncertainty.^23,27,71^ In addition, policies should also include specific measures to protect mothers and new-borns during periods of high environmental vulnerability.^59,64^ This may involve strategically relocating community clinics and improving family planning services in areas prone to displacement or affected by climate-related events.^65^ It is also crucial to expand the coverage and benefits of maternity allowance to improve access to and quality of maternal care in communities impacted by displacement and other adverse weather events.^27,65^ Furthermore, it is also recommended to expand access to family planning services and assisted reproductive technologies,^49,65^ by providing subsidised or comprehensive insurance coverage for contraceptives and fertility treatment, particularly in vulnerable areas.^84,85^

Another key aspect of policy responses to strengthen reproductive justice amid the climate crisis is ensuring effective resource planning and adequate investment in public health infrastructure. This includes guaranteeing that facilities are equipped and prepared for extreme weather conditions and ensuring that medical personnel are trained to respond to these critical events.^86^ It is also imperative to develop and implement resettlement policies for communities in areas at high risk of climate-related disasters.^65^ These policies should ensure that reproductive health services are integrated into resettlement plans, ensuring the availability of antenatal and postnatal care and access to emergency services during childbirth.^65^ Together, these measures can create a framework of resilience and adaptability that will enable communities to meet the challenges of climate change more safely and effectively, safeguarding reproductive rights amidst environmental degradation.

### Limitations

While this systematic review provides robust empirical evidence on the impact of the climate crisis on reproductive justice, it also has some limitations. First, since only empirical studies using primary or secondary data published in English between 2014 and 2024 were included, it is possible that we excluded relevant articles published before 2014 in languages other than English. Second, since we only included studies that explicitly referred to the climate crisis or climate change in the title, it is possible that relevant studies addressing the impact of particular climate-related events on reproductive justice were not considered. Lastly, we included empirical studies on climate crisis and reproductive justice that used both quantitative and qualitative methodological approaches. While this approach allowed for a more comprehensive assessment of the impact of the climate crisis on the creation and sustainability of human life, it also poses some limitations to the generalisation of the findings.

More studies are needed to further understand how the climate crisis is disrupting reproductive justice. As identified in this review, there is a critical gap of empirical evidence on the impact of climate-related events on the right not to have children. Consequently, it is vital that future research examines the impact of the climate crisis on contraception^87^ and abortion^88^ through the reproductive justice framework. Given current worldwide trends of fertility decline, it is also important that future research explores the impact of climate change on infertility^10,15^ and reproductive technologies^8^ through the reproductive justice framework. Overall, this approach will contribute to further elucidating not only how the climate crisis is affecting reproductive lives, but also how to enhance human rights and social justice amid environmental degradation and contribute to policies ensuring more sustainable reproductive futures.

As most existing research has focused on high-income countries, it is essential to further investigate the effects of the climate crisis on reproductive justice in low- and middle-income countries, where populations are particularly vulnerable to environmental degradation.^89^ Lastly, acknowledging that the climate crisis disproportionately impacts disadvantaged groups – including women, pregnant individuals, young children, and people with disabilities^3^ – future research should adopt an intersectional approach to expose how the sexual and reproductive rights of these and other communities are particularly vulnerable to long-term patterns of environmental change.

## Author contributions

Conceptualisation: MYD. Data curation: MYD, VGA, LW. Formal analysis: VGA. Investigation: MYD, VGA. Methodology: MYD. Resources: MYD. Supervision: MYD. Writing – original draft: MYD, VGA, LW. Funding acquisition: MYD. Project administration: MYD. Visualisation: VGA.

## Supplementary Material

Search strategy

## Supplementary file

Full electronic search strategy for all sources consulted.
