# Supplementary material for: From conception to care: a systematic review of the impact of the climate crisis on reproductive justice
Source: Sex Reprod Health Matters. 2025 Oct 22;33(1):2576365. doi: 10.1080/26410397.2025.2576365 (PMC12707084; doi:10.1080/26410397.2025.2576365)
Supplement: Search strategy [file ZRHM_A_2576365_SM0330.docx]

**Search strategy**

TI=Searches article titles. Title refers to the title of a journal article, proceedings paper, book, or book chapter. Note: To search for the title of a journal, select the Publication Title field.

filters= articles; 2014-2024; english

Keywords used for title search: “climate crisis” OR “climate change” AND “reproduction” OR “contraception” OR “fertility” OR “childbearing” OR “infertility” OR “pregnancy” OR “abortion” OR “birth” OR “care”.

Advanced Search: (TI=(climate crisis OR climate change)) AND TI=(reproduction OR contraception OR fertility OR childbearing OR infertility OR pregnancy OR abortion OR birth OR care).

Filters:

- Year: 2014 - 2024 [refine]
- Document type: Article [refine]
- Languages: English [refine]

**PubMed**

- Date: 06.06.2024
- Keywords used for title search: “climate crisis” OR “climate change” AND “reproduction” OR “contraception” OR “fertility” OR “childbearing” OR “infertility” OR “pregnancy” OR “abortion” OR “birth” OR “care”.
- Advanced Search: ((climate crisis[Title]) OR (climate change[Title])) AND (reproduction[Title] OR contraception[Title] OR fertility[Title] OR childbearing[Title] OR infertility[Title] OR pregnancy[Title] OR abortion[Title] OR birth[Title] OR care[Title])
- Results by year: 2014- 2024
- Languages: English [refine]
- Document type: Article [refine]

**Web of Science (Wos)**

- Date: 06.06.2024
- Keywords used for title search: “climate crisis” OR “climate change” AND “reproduction” OR “contraception” OR “fertility” OR “childbearing” OR “infertility” OR “pregnancy” OR “abortion” OR “birth” OR “care”.
- Advanced Search: (TI=(climate crisis OR climate change)) AND TI=(reproduction OR contraception OR fertility OR childbearing OR infertility OR pregnancy OR abortion OR birth OR care).
- Refined By: 2024 or 2023 or 2022 or 2021 or 2020 or 2019 or 2018 or 2017 or 2016 or 2015 or 2014.
- Languages: English [refine]
- Document types: Articles [refine]

**Scopus**

- Date: 06.06.2024
- Keywords used for title search: “climate crisis” OR “climate change” AND “reproduction” OR “contraception” OR “fertility” OR “childbearing” OR “infertility” OR “pregnancy” OR “abortion” OR “birth” OR “care”.
- Search strategy: Search within= Article title; Search documents= “climate crisis” OR Search within= Article title; Search documents= “climate change” AND Search within= Article title; Search documents= “reproduction” OR Search within= Article title; Search documents= “contraception” OR Search within= Article title; Search documents= “fertility” OR Search within= Article title; Search documents= “childbearing” OR Search within= Article title; Search documents= “infertility” OR Search within= Article title; Search documents= “pregnancy” OR Search within= Article title; Search documents= “abortion” OR Search within= Article title; Search documents= “birth” OR Search within= Article title; Search documents= “care”
- Year: 2014 - 2024 [refine]
- Document type: Article [refine]
- Languages: English [refine]

**GreenFILE**

- Date: 06.06.2024
- Keywords used for title search: “climate crisis” OR “climate change” AND “reproduction” OR “contraception” OR “fertility” OR “childbearing” OR “infertility” OR “pregnancy” OR “abortion” OR “birth” OR “care”.
- Advanced Search: TI (climate crisis or climate change) AND TI (reproduction OR contraception OR fertility OR childbearing OR infertility OR pregnancy OR abortion OR birth OR care)
- Publication Date (1) > Custom range: 01/01/2014 > 06/06/2024 [refine]
- Databases (1) > GreenFILE [refine]
- Source Types (1) > Academic Journals [refine]
- Language > English [refine]

**ProQuest Central**

- Date: 06.06.2024
- Keywords used for title search: “climate crisis” OR “climate change” AND “reproduction” OR “contraception” OR “fertility” OR “childbearing” OR “infertility” OR “pregnancy” OR “abortion” OR “birth” OR “care”.
- Advanced Search: ((climate crisis[Title]) OR (climate change[Title])) AND (reproduction[Title] OR contraception[Title] OR fertility[Title] OR childbearing[Title] OR infertility[Title] OR pregnancy[Title] OR abortion[Title] OR birth[Title] OR care[Title])
- Publication date: 2014- 2024
- Languages: English [refine]
